# Diabatic and adiabatic transitions between Floquet states imprinted in coherent exciton emission in monolayer WSe_2_

**DOI:** 10.1126/sciadv.abq7281

**Published:** 2022-12-21

**Authors:** Kento Uchida, Satoshi Kusaba, Kohei Nagai, Tatsuhiko N. Ikeda, Koichiro Tanaka

**Affiliations:** ^1^ Department of Physics, Graduate School of Science, Kyoto University, Sakyo-ku, Kyoto 606-8502, Japan.; ^2^ Institute for Solid State Physics, University of Tokyo, Kashiwa, Chiba 277-8581, Japan.; ^3^ Institute for Integrated Cell-Material Sciences, Kyoto University, Sakyo-ku, Kyoto 606-8501, Japan.

## Abstract

Floquet engineering is a promising way of controlling quantum system with photon-dressed states on an ultrafast time scale. So far, the energy structure of Floquet states in solids has been intensively investigated. However, the dynamical aspects of the photon-dressed states under ultrashort pulse have not been explored yet. Their dynamics become highly sensitive to the driving field transients, and thus, understanding them is crucial for ultrafast manipulation of a quantum state. Here, we observed the coherent exciton emission in monolayer WSe_2_ at room temperature at the appropriate photon energy and the field strength of the driving light pulse using high-harmonic spectroscopy. Together with numerical calculations, our measurements revealed that the coherent exciton emission spectrum reflects the diabatic and adiabatic dynamics of Floquet states of excitons. Our results provide a previosuly unexplored approach to Floquet engineering and lead to control of quantum materials through pulse shaping of the driving field.

## Teaser

Observing a signature of quantum mechanical jump between photon-dressed states in excitons through high harmonic emission.

## INTRODUCTION

**Fig. 1.**
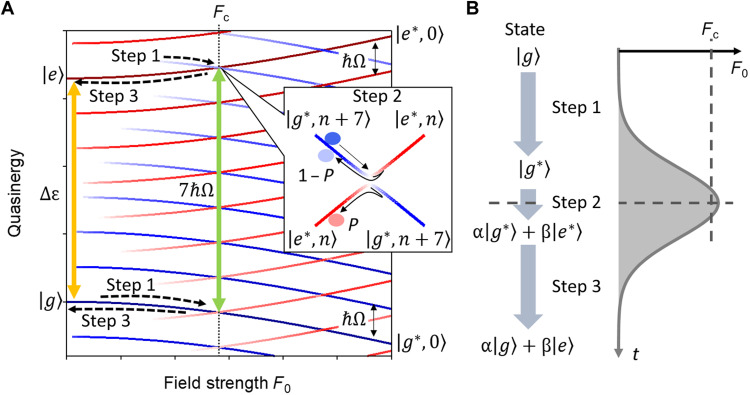
Temporal evolution of Floquet state in a two-level system. (**A**) Schematic diagram of Floquet energy bands as a function of field strength *F*_0_. Here, the solution of time-dependent Schrödinger equation is defined by $|i^{*}\rangle =\exp(-i\varepsilon_{i}^{*}t/$ℏ$)\sum |i^{*},l\rangle \exp(il\Omega t)$, where $\varepsilon_{i}^{*}$ is quasienergy and ∣i^*^, *l*⟩ is the *l*th sideband state of Floquet state ∣i^*^⟩. Blue (red) lines represent ladder-like energy levels of the ground (excited) Floquet state separated by a driver photon energy of ℏΩ. The depth of color represents the magnitude squared of the Floquet sidebands given by ⟨i^*^, *l* ∣ i^*^, *l*⟩ with logarithmic scale. The inset shows an expanded view of the avoided crossing between the Floquet ground and excited states. Here, the label of Floquet states (i^*^ = g^*^ or e^*^) is assigned in diabatic picture. (**B**) Schematic evolution of quantum state under slowly varying field amplitude *F*_0_(*t*). Step 1. The initial ground state ∣*g*⟩ transforms into a Floquet state ∣g^*^⟩ and shows an energy shift with increasing *F*_0_. Step 2. At the first avoided crossing, a diabatic transition from ∣g^*^⟩ to ∣e^*^⟩ occurs, as shown in the inset. Step 3. The created superposition between the ground and excited Floquet states (α∣g^*^⟩ + β∣e^*^⟩) oscillating at 7Ω adiabatically transforms into a superposition of bare state (α∣*g*⟩ + β∣*e*⟩) oscillating at Δε/ℏ, where Δε = ε_e_ − ε_g_.

Intense light waves are a useful tool for transforming functionalities of electronic systems on an ultrafast time scale through nonlinear light-matter interactions (*1*–*4*). When the light wave is periodic in time (i.e., an ideal continuous wave), the driven electronic system is well described by the Floquet states (*5*). They are “photon-dressed states” reflecting the effective Hamiltonian of the driven system, and their physical properties can be tuned by parameters such as the amplitude, frequency, and polarization of the light wave. Such a tuning capability has given rise to the concept of Floquet engineering, one of the most promising candidates for ultrafast manipulation of quantum systems (*6*–*9*). The Floquet states under periodic driving *F*(*t*) = *F*_0_ cos Ω*t* are characterized by a ladder-like energy structure given by ε^*^(*F*_0_, Ω) + *n*ℏΩ (ε^*^: quasienergy, *n*: integer, Ω: driver angular frequency) according to the Floquet theorem (Fig. 1A). Such a quasienergy structure has been observed using several ultrafast experimental techniques in solids (*10*–*12*).

However, the above Floquet picture assumes a continuous-wave driving (*F*_0_ = const.) and hence is approximate in real experiments, where ultrashort pulses are usually used to yield intense fields. Instead, for finite-width pulses, the Floquet states arise only instantaneously at the time-dependent amplitude [*F*_0_ = *F*_0_(*t*)] (*13*–*16*). These instantaneous Floquet states and their quasienergies gradually change along the pulse envelope *F*_0_(*t*), and the quantum state of the system either adiabatically follows or diabatically jumps between them. For the ultimate Floquet engineering, it is crucial to control those dynamics of the Floquet states during the pulse. Nevertheless, direct or indirect signatures of them have not yet been experimentally obtained in solids because of the fast dephasing of electrons in solids and the necessity of a huge quasienergy shift comparable to driving photon energy for observable effects. The lack of an established method that can access coherent electron dynamics during the pulse duration also makes it challenging to observe the dynamics of Floquet states.

Electronic systems driven by intense light waves emit coherent electromagnetic radiation, which entails the dynamical information of the system and can hence be indirect evidence of instantaneous Floquet states. For example, short-time dynamics that repeat with the driving field cycle induce high harmonic generation (HHG), i.e., radiation with integer multiples of the driving photon energy (*17*–*22*). Such subcycle dynamics are usually analyzed in terms of diabatic and adiabatic processes on the unperturbed (i.e., “undressed”) state basis as well as the single Floquet eigenstate using the Floquet picture (*7*, *23*). Meanwhile, in the dynamics longer than the driving field cycle during the pulse, the adiabatic and diabatic dynamics among instantaneous Floquet states discussed above can be relevant, strongly modifying high harmonics properties or even inducing novel nonlinear optical phenomena beyond high harmonic emissions.

Here, we propose that diabatic transition between Floquet states can be effectively induced by setting the appropriate driving photon energy and field strength, and the nonlinear emissions from the driven system can probe the dynamics of Floquet states. Let us consider the simplest model embodying this concept: a two-level system excited by an intense laser pulse showing transitions between two instantaneous Floquet states. Figure 1A shows the quasienergy structure of the two-level system as a function of the field amplitude *F*_0_. We assume that the envelope of the driving field *F*_0_(*t*) varies slowly enough that the Floquet states are useful instantaneous basis: The electronic system at *t*_0_ can be described by the Floquet state with lightwave amplitude *F*_0_(*t* = *t*_0_). The validity of this assumption was experimentally confirmed for short pulses (*24*). When the driving field amplitude is sufficiently weak, the transition probability as a function of incident photon energy has a local maximum at the well-known multiphoton resonance condition. When the field amplitude is strong, however, the energy levels shift to be quasienergies, and the multiphoton resonance condition reads

$${\Delta \varepsilon }^{*}(\Omega ,F_{0})\approx n\hbar \Omega$$ (1)

where Δε^*^ is the quasienergy difference between two Floquet states. This condition means that the two quasienergies approach to show an avoided crossing as illustrated in the inset of Fig. 1A, where the diabatic transition between the two Floquet states efficiently occurs.

Because the quasienergy depends on the driving field amplitude, the resulting transition probability between two Floquet states intricately depends on the time profile of the field amplitude *F*_0_(*t*) (*13*, *14*, *16*). As *F*_0_ increases, the initial ground state ∣*g*⟩ adiabatically transforms into the Floquet state ∣g^*^⟩, approaching the first avoided crossing with the Floquet state ∣e^*^⟩ (Step 1 in Fig. 1). For brevity, we call ∣g^*^⟩ and ∣e^*^⟩ the Floquet ground and excited states, respectively. When the peak driving field amplitude is below the critical amplitude *F*_c_ satisfying Eq. 1, the system does not reach the first avoided crossing and, thus, returns to the original ground state after the pulse duration without making a transition. On the other hand, when the peak electric field strength reaches the critical field *F*_c_, a diabatic transition from the Floquet ground to an excited states occurs with transition probability *P*, as predicted by solving the Floquet-Landau-Zener problem (Step 2 in Fig. 1) (*13*, *14*, *16*). This transition creates a superposition (coherence) between two Floquet states oscillating at Δε^*^/ℏ + *n*Ω. After the transition, as the field strength *F*_0_ gradually decreases, the created coherence adiabatically evolves into the bare state coherence (Step 3 in Fig. 1). This means that high harmonic emissions oscillating at integer multiples of the driver frequency are gradually converted into a coherent emission resonant with the transition of the original system as a signature of the diabatic and adiabatic evolution of the Floquet states.

Note that incoherent emissions resonant with the transition energy often observed after the nonresonant and intense driving (*17*, *18*) are different from the above coherent ones. Luminescence from carriers created through a strong field-induced tunneling process and then relaxed into the band edge can explain the incoherent emission without the help of the Floquet state picture. On the other hand, coherent emissions are not allowed except at integer multiples of the driving frequency in perturbative nonlinear optics (*25*) and in nonperturbative nonlinear optics due to intercycle interference. Strong and dynamical alteration of the energy structure of the driven system during the pulse duration, which is the manifestation of the Floquet engineering, plays a crucial role in activating the forbidden coherent emission. To observe the above phenomena in solids, whose typical energy scale is on the order of electron volts, we need a short mid-infrared (MIR) pulse whose field strength is typically above 1 MV cm^−1^. The transition probability depending on *F*_0_(*t*) provides us with a knob for Floquet engineering, i.e., manipulation of the population of Floquet states.

To demonstrate the dynamical control of Floquet states in solids, we focused on excitons in semiconducting transition metal dichalcogenides (TMDCs). An exciton is a bound state of an electron-hole pair formed by Coulomb attraction. Because of it being a bound state, its dephasing time is much longer than that of the unbound electron-hole pairs. In particular, monolayer TMDCs are an ideal two-dimensional system with a direct bandgap and host excitons with a large binding energy of several hundred milli–electron volts, which are stable even at room temperature (*26*–*30*). An atomically thin layer also enables us to obtain intrinsic nonlinear optical signals without a nonlinear propagation effect (*31*), which strongly modifies nonlinear optical properties. Under strong infrared light driving, excitons in monolayer TMDCs form a Floquet state and show large energy shifts called the optical Stark shift and Bloch-Siegert shift (*32*–*34*). In addition, efficient HHG and the related phenomena have been observed in a group of monolayer TMDCs (*35*–*39*). Thus, stable excitons and their strong coupling to light provide us an ideal platform to observe the dynamical aspects of the Floquet states.

## RESULTS

**Fig. 2.**
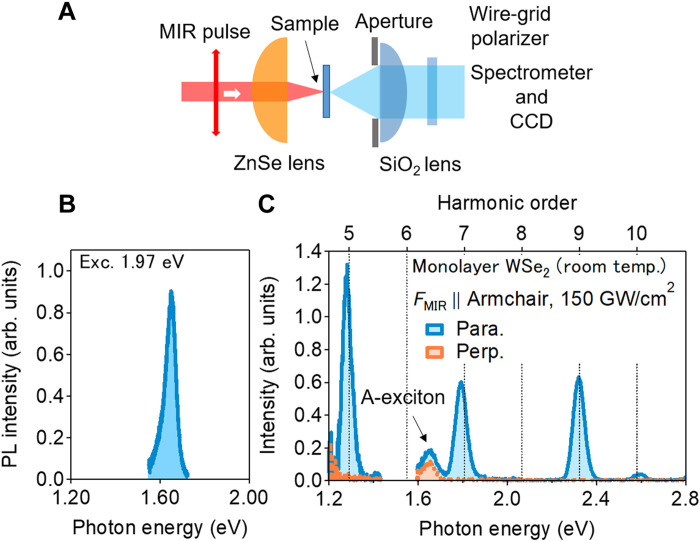
Observation of nonlinear emission process in monolayer WSe_2_ at room temperature. (**A**) Experimental setup of nonlinear emission measurement. The spot size of the MIR pulse at the focal point is estimated to be 30 μm (full width at half maximum). All the experiments were performed at room temperature in an ambient condition. (**B**) Photoluminescence spectrum from the monolayer WSe_2_ sample. (**C**) Typical nonlinear emission spectra from the monolayer WSe_2_ sample. Light blue (orange) indicates the spectrum component whose polarization is parallel (perpendicular) to the MIR polarization. Here, the MIR polarization direction is along the armchair direction of monolayer WSe_2_, which is determined by the selection rule of HHG (*35*, *36*). MIR intensity is estimated to be 170 GW cm^−2^.

We prepared a monolayer WSe_2_ sample using the mechanical exfoliation technique and attached it to a quartz substrate (see section S1). Figure 2A shows a schematic diagram of the experimental setup. We used an intense linearly polarized MIR driver to excite the sample. The MIR photon energy is set to be 0.26 eV so that the seventh harmonic energy (1.81 eV) is above the A-exciton resonance at room temperature (1.65 eV), which was confirmed by making a photoluminescence measurement (Fig. 2B). The A-exciton resonance far from any integer multiples of the driving photon energies (harmonic emission energies) is suitable for observing the conversion of high harmonics into bare excitonic coherence. The maximum intensity at the focal point is set to be 170 GW cm^−2^ in air, corresponding to an electric field strength of 11 MV cm^−1^. Under such a strong electric field, the A-exciton transforms into a Floquet state, and its resonance (quasienergy) is expected to be shifted higher, on the order of 0.1 eV for the peak intensity (*32*–*34*, *40*). This huge quasienergy shift enables the seven-photon resonance condition to be satisfied for dressed A-excitons (*n* = 7 for Eq. 1). The pulse width was estimated to be 60 fs (about six-cycle pulse) using the sum-frequency generation technique (*38*). In accordance with previous experimental and theoretical investigations (*24*, *16*), we assumed that Floquet states are a useful instantaneous basis in this experimental condition. The emissions induced by the driver were collected using an ultraviolet grade (UV) silica lens with an aperture to resolve the divergence of the emission beam. The polarization of the collected emissions was resolved using a wire grid polarizer, and emission spectra were then detected using a spectrometer equipped with a charge-coupled device (CCD) camera. All the experiments were performed at room temperature in ambient conditions.

Figure 2C shows typical emission spectra from monolayer WSe_2_ for an MIR electric field in the armchair direction. We observed clear high harmonics signals at *n*ℏΩ. The polarization of the high harmonics was parallel to the incident MIR polarization, as determined from the crystal symmetry (*35*, *36*). In addition, we observed an emission at the A-exciton resonance with parallel and perpendicular components. One possible origin of the resonant emission is incoherent emission from excitons. So far, there have been several reports on luminescence from incoherent excitons created by intense terahertz or MIR pulses associated with high harmonics (*17*, *18*). However, in our experiment, the intensity of the parallel component was larger than that of the perpendicular one. WSe_2_ hosts the circular polarization selection rule based on the valley degree of freedom (*28*, *41*, *42*). Therefore, the observation of partially linearly polarized exciton emissions indicates that the superposition of two valley states (K and K′ valleys) is partially preserved. One possible origin is the excitonic coherence created by the dynamics of the Floquet state. The other is the so-called valley-coherence luminescence (*43*, *44*), where the excitonic coherence is lost, but superposition of the two valley states is maintained.

**Fig. 3.**
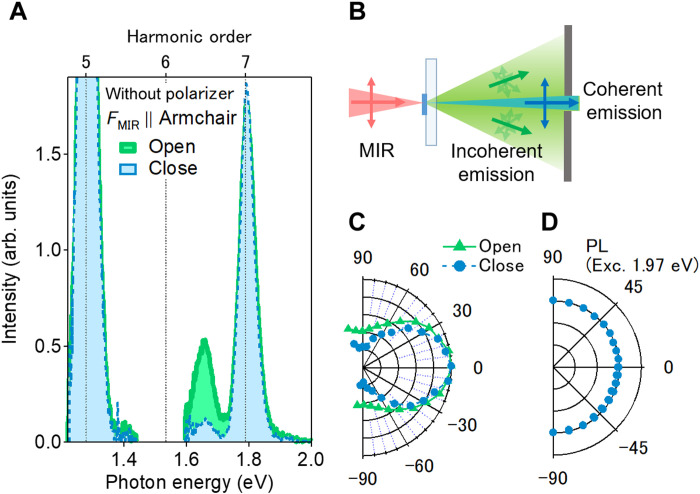
Characteristics of excitonic resonant emission. (**A**) Nonlinear emission spectra near the A-exciton resonance with open (green) and closed (light blue) aperture configurations. (**B**) Schematic exciton emission from the sample. (**C**) Polarization-resolved intensities of the excitonic resonant emission with open (green squares) and closed (blue circles) aperture configurations. Here, 0° is the direction parallel to the MIR polarization. (**D**) Polarization-resolved intensities of photoluminescence from A-exciton created by 1.97 eV picosecond laser excitation. Here, 0° is the direction parallel to the polarization of the excitation laser.

To confirm the existence of excitonic coherence, we have measured spatial coherence of the exciton emission. Coherent exciton emission should have a small beam divergence as observed in the spatially coherent signals such as the high harmonics, but luminescence (incoherent exciton emission) has a large beam divergence regardless of the space-time coherence of the MIR driver. Figure 3A shows the emission spectra with open- and closed-aperture configurations. Figure 3B shows a schematic setup for polarization-resolved exciton emission measurements with aperture. We collected emissions with a numerical aperture of 0.56 (0.06) for the open (closed) configuration. When the emission has coherence reflecting the wavefront of the driving field, the beam divergence becomes small compared with that from an incoherent emission such as luminescence. For example, because HHG is a coherent optical process, the divergence of HHG signals is small enough to detect all the signals even in the closed-aperture configuration. On the other hand, in the exciton emission, where there is also a contribution from incoherent emission, the intensity decreases when the aperture is closed.

Here, we define the degree of linear polarization (DOLP) as (*I*_para_ − *I*_perp_)/(*I*_para_ + *I*_perp_), where *I*_para_ and *I*_perp_ are the exciton emission intensities of the parallel and perpendicular components, respectively. By closing the aperture, the DOLP of the exciton emission should increase if there are contributions from both coherent and incoherent excitons. Figure 3C shows the polarization states of exciton emissions in open-aperture (orange squares) and closed-aperture (blue circles) configurations. By closing the aperture, the DOLP changes from 0.4 to 0.7, indicating the existence of the excitonic coherence. The above hypothesis is supported by the results of a similar experiment we conducted on thin-layer bulk WSe_2_. Because the luminescence efficiency is strongly suppressed in the bulk WSe_2_ due to the indirect bandgap, we expected only coherent emissions from bulk WSe_2_. We found that the DOLP of the exciton emission was almost 1.0 in bulk WSe_2_ (see section S3 for a detailed discussion).

By performing photoluminescence measurement, we also ruled out any valley coherence luminescence contribution to the linearly polarized exciton emission, which has been reported in monolayer TMDCs mainly at low temperatures (*43*, *44*). Figure 3D shows the polarization state of luminescence measured by a picosecond laser excitation at 1.97 eV (630 nm) at room temperature. We could not find any features of valley coherence within the signal-to-noise ratio, indicating that the emissions with high DOLP originated only from the excitonic coherence at room temperature. Therefore, we can distinguish the contribution of the excitonic coherence by subtracting the perpendicular components from the parallel ones.

## DISCUSSION

**Fig. 4.**
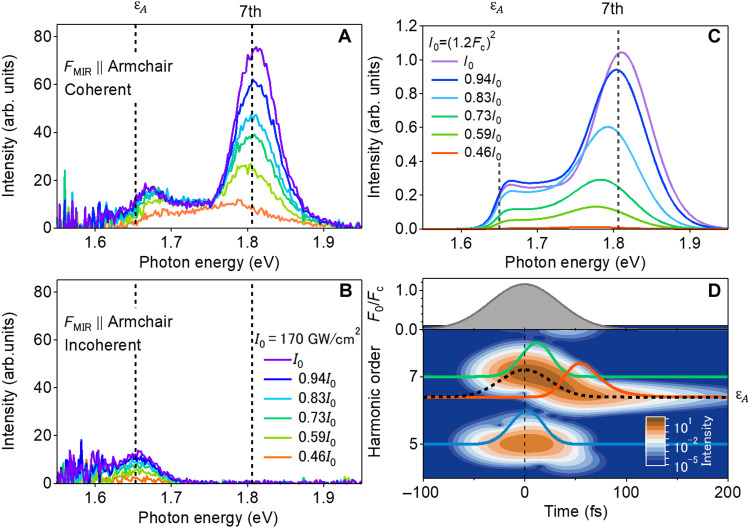
Spectral evolution of the excitonic resonant emission depending on MIR intensity. (**A** and **B**) Measured nonlinear emission spectra of coherent (A) and incoherent (B) components near the A-exciton resonance with MIR intensities from 0.46 *I*_0_ to *I*_0_. The MIR polarization is along the armchair direction. (**C**) Computed nonlinear emission spectra near the resonance in the two-level model. The detailed conditions of the calculation are described in section S5-6. (**D**) Top: Temporal profile of the driving field amplitude. Bottom: Emission intensity of the two-level model as a function of time and harmonic order obtained using Gabor transformation. Here, the time window of the Gabor transformation is 20 fs. Blue, orange, and green solid lines show the emission intensities at the fifth harmonic, the excitonic resonance, and the seventh harmonic, respectively. The maximum intensities are normalized for clarity. The black dashed line represents the calculated quasienergy of the dressed A-exciton in the two-level model. Here, the quasienergy at time *t* is evaluated using the field amplitude of *F*_0_(*t*).

Figure 4 (A and B) shows the distinguished coherent and incoherent emission spectra near the excitonic resonance. Compared with the incoherent emission spectrum, the coherent emission peak is slightly blue-shifted and has a spectral component between the exciton resonance and the seventh harmonic. In addition, we can see spectral distortion in the seventh harmonic. The seventh harmonic peak shows a red shift just below the intensities where strong coherent exciton emissions emerge. Such a complex spectral change depending on MIR intensity was not seen in the fifth harmonic (see section S4) and suggests the effect of the Floquet state dynamics depicted in Fig. 1.

We simulated the exciton response under nonresonant driving by numerically solving a simple two-level model considering an electron-hole vacuum and the 1s level of the A-exciton in monolayer WSe_2_ (see section S5-1 for a detailed description). Figure 4C shows the calculated emission spectra near the excitonic resonance with several field amplitudes near the critical field *F*_c_. Because the two-level model is a rough approximation neglecting higher excitonic states, it is nontrivial whether coherent exciton dynamics can be mapped on to simple Floquet dynamics of the two levels depicted in Fig. 1. However, the simulation qualitatively reproduces the spectral features of the observed coherent exciton emissions including its slight blue shift. In addition, the simulated seventh-order harmonics show a spectral modification similar to that of the experiments, i.e., an energy shift depending on driver intensity. This indicates that the observed excitonic response can be regarded as that of the simple two-level system depicted in Fig. 1: Intense MIR driving induces a huge A-exciton resonance shift of ~0.15 eV at maximum intensity, creating excitonic coherence as a signature of the diabatic transition between the Floquet states. Note that we also solved the linearized semiconductor Bloch equation for the two-band model, which takes higher excitonic states or continuum states into account. We qualitatively obtained the same results when the transition of the 1s exciton to higher excitonic states is not salient (see section S5-6 for details) (*45*–*47*).

To extract the dynamics of the nonlinear emissions, we performed a Gabor transformation on the calculated emission (interband polarization), as shown in Fig. 4D. The Gabor transformation enables us to track the temporal evolution of the energy of the nonlinear emissions with finite temporal and energy resolutions. The fifth harmonic signal (the blue line), whose emission energy is below the exciton resonance, shows an almost instantaneous response to the envelope of the driving field *F*_0_(*t*) (gray area in the top panel), which is consistent with the conventional HHG response below the bandgap energy (HHG originating from Kerr-type nonlinearity) (*48*).

On the other hand, the seventh harmonic signal (the light green line in Fig. 4D) is delayed relative to the envelope of the driving field *F*_0_(*t*). Because the electronic polarization is delayed when its frequency is nearly resonant with a certain transition (see section S7), the delay in the seventh harmonic indicates its resonance with the dressed A-excitons. At the bare excitonic resonance energy, the amplitude of the emission grows as the driving field amplitude decreases (the orange line in Fig. 4D). This evolution reflects the energy shift of nonlinear emission from that of the seventh harmonic to the bare excitonic resonance. This energy shift clearly follows the time evolution of the quasienergy of the dressed exciton (the black dashed line), which means that the Floquet state transformed adiabatically into the bare exciton state under our experimental conditions. The adiabatic dynamics of the Floquet state leads to the characteristic of coherent exciton emission in Fig. 4 (A and C): a slight blue shift of the spectral peak, a spectral component between the seventh harmonic and the excitonic resonance.

The simulations revealed that the pulse width of the driver and the dephasing time of the exciton are crucial factors to manipulate diabatic and adiabatic transition between the Floquet states. If a dephasing time is much shorter than the falling edge of the driver pulse (30 fs in our experimental setup), the decoherence process strongly suppresses the conversion of the seventh harmonics into the excitonic signal (see section S5-5 for a detailed discussion). An exciton dephasing time of at least 20 fs is required to obtain an exciton signal comparable to the seventh harmonic. By using the spectral width of the A-exciton resonance in a sample from the same batch that was encapsulated with hexagonal boron-nitride flakes, which strongly suppress inhomogeneous broadening, we estimated the homogeneous broadening due to phonon scattering to be 25 meV at room temperature in the sample of this experiment (*49*, *50*). This value corresponds to the dephasing time of 26 fs and is consistent with the simulation value reproducing the experimental results. This much longer dephasing time of the exciton compared with unbound electron-hole pairs enables us to observe a clear signature of the dressed exciton dynamics in solids even at room temperature.

**Fig. 5.**
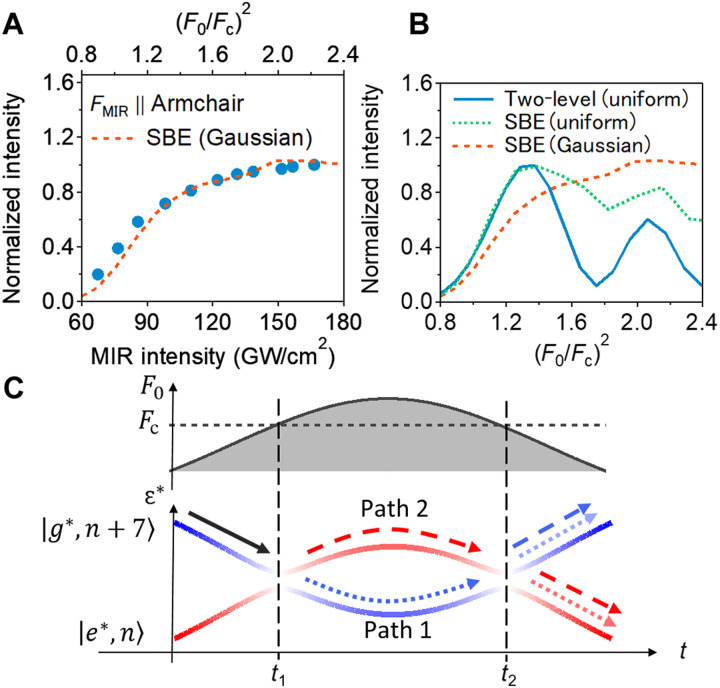
Field strength dependence of the excitonic resonant emission signal. (**A**) Normalized coherent exciton emission intensity as a function of MIR intensity for electric field parallel to armchair directions (blue circles). Top axis represents the relative square of field strength to the critical field *F*_c_, which has best agreement with the simulation result including Gaussian distribution of the field and the contribution of higher excitonic levels (orange dashed curve). (**B**) Computed nonlinear emission intensity as a function of the square of the driving field strength. Blue solid curve represents the results using the two-level model with the uniform field distribution. Green dotted and orange dashed curves represent those using the semiconductor Bloch equation, which takes into account higher excitonic states, with uniform and Gaussian field distributions, respectively. (**C**) Schematics of Floquet-Landau-Zener interference. Top: Temporal profile of the driving field amplitude. Bottom: Temporal evolution of quasienergies of instantaneous Floquet states. Path 1 (dashed arrows): Quantum trajectories that show the adiabatic evolution at the first passage of the avoided crossing (*t*_1_). Path 2 (dotted arrows): Quantum trajectories that show the diabatic transition at *t*_1_. The interference between these two paths occurs after the second passage of the avoided crossing at *t*_2_.

Last, we address the driving field strength dependence of the coherent emission signal. Figure 5A shows the excitonic-coherence intensities as a function of MIR intensity for the electric field parallel to the armchair direction. The coherent exciton signal is strongly saturated with increasing MIR intensity especially above 140 GW cm^−2^ (corresponding to 0.83 *I*_0_ in Fig. 4A). In the simple two-level model, the signal also shows the saturation just after the critical field strength *F*_c_ and oscillates as the field becomes stronger (blue solid curve in Fig. 5B). This saturation and oscillation of signal are signatures of the Floquet-Landau-Zener interference (*16*). When the peak driving field strength is sufficiently higher than the critical field *F*_c_, there are two passages of the same avoided crossing point at *t*_1_ and *t*_2_, and this causes the quantum interference between Path 1 and Path 2 in Fig. 5C. With an increase of the field strength, the interference effect gradually changes from the constructive one to the destructive one, resulting in the oscillation of the coherent excitonic signal. In our experiment, we could not observe oscillation behavior in coherent excitonic signals. The discrepancy between the experiment and two-level simulation comes from the neglected factors in the simple two-level model. One factor is the contribution of higher excitonic states. Strong field driving causes the transition from the 1s exciton to higher excitonic states (*51*), which can be regarded as the increase in the effective dephasing rate for 1s excitonic coherence (*52*). This field-induced dephasing weakens Floquet-Landau-Zener interference, i.e., oscillation of signal depending on the field (green dotted curve in Fig. 5B). Another factor is the inhomogeneous MIR field distribution within the MIR spot. The averaging of the signal over the MIR spot smooths out the intrinsic oscillation behavior (*53*). Considering these two factors, we can reproduce the experimental results well, as shown in the orange dashed curve in Fig. 5B (see section S6 for the detailed discussion).

In summary, we demonstrated that coherent exciton emissions are induced by intense MIR driving in monolayer WSe_2_ at room temperature as a signature of the Floquet state dynamics. Coherent exciton emissions far away from the harmonic emission energies indicate a dynamical energy structure change exceeding 0.1 eV, a diabatic transition between the Floquet states of the vacuum and the 1s A-exciton, and an adiabatic transformation of the Floquet states into bare A-exciton states. Our study provides a new method to access the Floquet state and indicates that monolayer TMDC is a fascinating platform for realizing coherent control of quantum properties on an ultrafast time scale. Time- and energy-resolved measurements of the coherent emission using ultrashort pulses will enable direct observation of Floquet state dynamics, as depicted in Fig. 4D. For a peak driving field amplitude much higher than in our experimental conditions, multiple avoided crossings between Floquet states are expected to be involved in the temporal evolution of the system. In this situation, the transition probability highly depends on the shape of the driving field pulse, owing to Floquet-Landau-Zener interference (*16*). Therefore, pulse shaping of an intense driving field with control of light helicity might provide a knob for ultrafast control of the valley degree of freedom in monolayer TMDCs (*37*, *54*).

## METHODS

The laser source is a Ti:Sapphire regenerative amplifier (pulse width: 35 fs, pulse energy: 7 mJ, center wavelength: 800 nm, repetition rate: 1 kHz). Part of the laser output was used for optical parametric amplification to generate signal and idler outputs in the near-infrared region using Light Conversion TOPAS-C. MIR pulses (wavelength: 4800 nm) for nonlinear emission measurement were generated by using difference frequency mixing of signal and idler outputs. The signal and idler outputs were then blocked by a long-pass filter (cutoff wavelength: 4000 nm). The MIR polarization angle and intensity were controlled by two wire grid polarizers (Thorlabs, WP25M-IRA) and a liquid crystal variable retarder (Thorlabs, LCC1113-MIR). The MIR pulses were focused onto the sample with normal incidence using a ZnSe lens (focal length: 62.5 mm). The group delay dispersion due to the above optics was compensated by inserting a pair of CaF_2_ plate. The spot size at the focal point was estimated to be 60 μm in full width at half maximum using knife edge measurement, and the pulse width is estimated to be 60 fs using the sum frequency mixing of the fundamental and the MIR pulses in monolayer MoS_2_ (*38*). The transmitted nonlinear emissions were corrected by UV-fused silica lens (focal length: 50 mm). To evaluate the beam divergence of the nonlinear emission from the sample, we set an iris in front of the collection lens so that we can control the numerical aperture for beam collection. The collected emissions were polarization-resolved using a wire grid polarizer (Thorlabs, WP25M-UB), spectrally resolved by a spectrometer, and detected by a Si CCD camera. The crystal orientation of the sample was determined by the selection rule of HHG in monolayer WSe_2_ (*36*).

## Supplementary Materials

### This PDF file includes:

Sections S1 to S7 Figs. S1 to S14
