# Supplementary material for: Diabatic and adiabatic transitions between Floquet states imprinted in coherent exciton emission in monolayer WSe2
Source: Sci Adv. 2022 Dec 21;8(51):eabq7281. doi: 10.1126/sciadv.abq7281 (PMC9770970; doi:10.1126/sciadv.abq7281)
Supplement: Supplementary file 1 — Sections S1 to S7 Figs. S1 to S14 [file sciadv.abq7281_sm.pdf]

Science Advances
AAAS

Supplementary Materials for

Diabatic and adiabatic transitions between Floquet states imprinted in coherent exciton emission in monolayer WSe2

Kento Uchida *et al.*

Corresponding author: Kento Uchida, uchida.kento.4z@kyoto-u.ac.jp; Koichiro Tanaka, kochan@scphys.kyoto-u.ac.jp

Sci. Adv. **8**, eabq7281 (2022)
DOI: 10.1126/sciadv.abq7281

This PDF file includes:

Sections S1 to S7
Figs. S1 to S14

S1. Sample preparation.

We prepared monolayer WSe_2 by mechanical exfoliation of a commercial WSe_2 crystal grown by chemical vapor deposition (CVD), which was purchased from 2D semiconductors Inc. The monolayer was transferred onto the quartz substrate by dry transfer method. The sample was characterized by photoluminescence (PL) spectrum obtained by a commercial PL/Raman micro spectrometer (NanoFinder 30, Tokyo Instruments Inc.).

Figure S1 shows the optical image of the exfoliated WSe_2 sample. The size of the monolayer region is ~200 μm, which is sufficiently large for the MIR laser spot size of ~60 μm (white dashed circle).

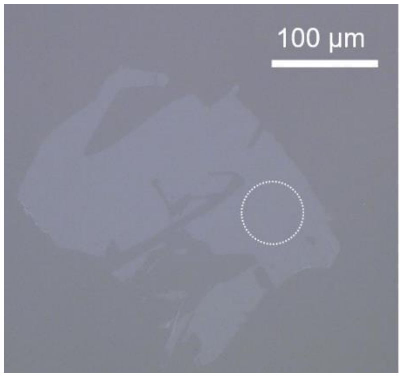

Figure S1: Optical image of the mechanically exfoliated monolayer WSe_2 sample. White dashed circle indicates the spot where MIR light was irradiated. The sample size is sufficiently larger than the MIR spot size, enabling us to obtain high signal-to-noise ratio signals.

S2. Polarization-resolved PL measurement setup.

The polarization-resolved PL measurement was performed in the backscattering configuration. We used a commercial supercontinuum white light source (WhiteLase, Fianium, total power: 4 W, repetition: 40 MHz, wavelength: 400~2400 nm). A home-built subtractive-mode double monochromator and a shortpass filter was used to obtain the excitation light with narrow linewidth. The excitation light was focused onto the sample by using an objective lens (Mitsutoyo, 20x, N.A.=0.28). The PL was collected by the same objective lens, polarization-resolved by using a film polarizer, spectrally resolved by a grating spectrometer, and detected by a liquid-nitrogen-cooled Si CCD camera.

S3. Nonlinear emission measurement in bulk WSe_2.

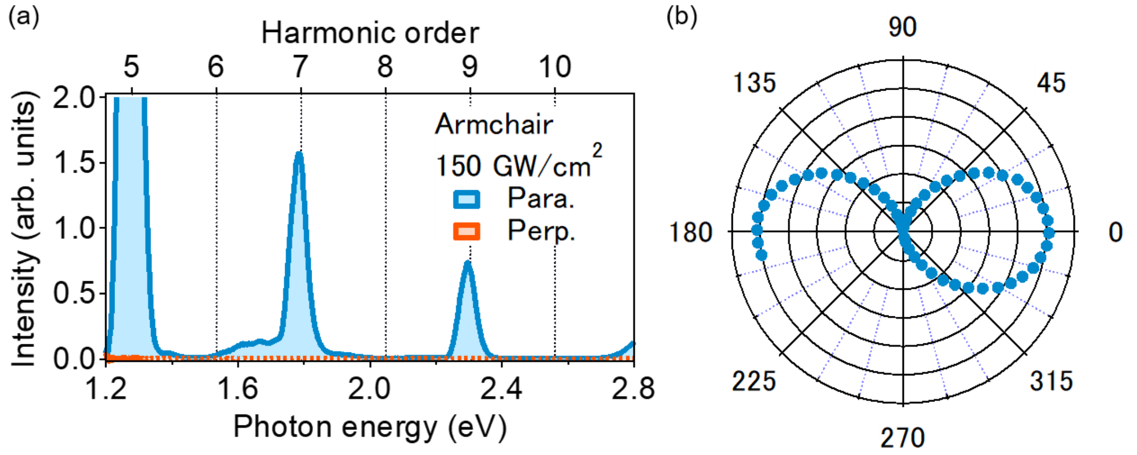

Figure S2: Nonlinear emission from bulk WSe_2. (a) Typical nonlinear emission spectra from the bulk WSe_2 sample. Light blue (orange) indicates the spectrum component whose polarization is parallel (perpendicular) to the MIR polarization. (b) Polarization-resolved intensities of the exciton emission. Here, 0 degree is the direction parallel to the MIR polarization.

Figure S2(a) shows typical emission spectra from bulk 2H-WSe_2 corresponding to that from monolayer in Fig. 2(c). Since 2H-WSe_2 has an inversion symmetry, there are no even-order harmonics, which observed in the monolayer sample. Similar to the monolayer sample, we observed emissions slightly below the seventh order harmonic (~ 1.6 eV), which is probably attributed to the resonant emission from direct excitons of bulk WSe_2. In bulk WSe_2, electrons excited at K-valley are scattered into Q-valley. This electron relaxation into Q-valley enables to strongly suppress the incoherent excitonic emissions, which hinder the coherent excitonic signals. Therefore, we can expect the only coherent exciton emission from the bulk sample. In fact, the perpendicular component of the nonlinear emission is negligible. Figure S2(b) shows the polarization state of the exciton emission. The emission is polarized parallel to incident MIR polarization, and its DOLP reaches 0.97. These results in bulk WSe_2 support our claim in the main text: excitonic coherence originated from Floquet state dynamics is involved in the exciton emission under non-resonant MIR field.

S4. MIR intensity dependence of the fifth harmonic.

Figure S3(a) shows the fifth harmonic spectra for several MIR intensities. Compared to the seventh harmonics shown in Fig. S3(b), spectral peak energy of the fifth harmonic is almost independent of MIR intensity. This suggests that the strong spectral modification of the seventh harmonic is caused by the Floquet state dynamics of A-exciton in monolayer WSe_2.

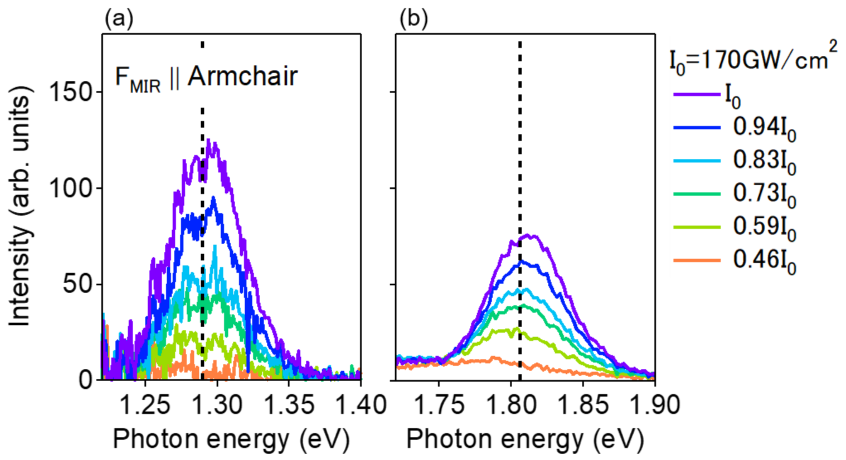

Figure S3: MIR intensity dependence of high harmonic spectrum. (a) The fifth harmonic spectra with several MIR intensities. (b) The seventh harmonic spectra with several MIR intensities.

S5. Numerical simulation of the excitonic response under intense MIR driving.

Here, we consider the excitonic response under the intense MIR field by numerically solving (i) the simplified two-level model and (ii) two-band model with the excitonic effect.

S5-1. Numerical simulation of the dynamics of two-level model.

Figure S4(a) shows the energy diagram of the two-level system. In this sub-section, for simplicity, we consider only the vacuum state for electron-hole pairs $|0\rangle$ and the 1s state of A-exciton $|1s\rangle$, and neglects higher excitonic states and

electron-hole continuum band. Then, Hamiltonian of the driven system can be described as follows:

$$H(t) = H_0 + H_I(t), \tag{S1}$$

$$H_0 = \begin{pmatrix} 0 & 0 \\ 0 & \varepsilon_{1s} \end{pmatrix}, \tag{S2}$$

$$H_I(t) = \begin{pmatrix} 0 & d_{1s}F(t) \\ d_{1s}F(t) & 0 \end{pmatrix}. \tag{S3}$$

Here, ε_{1s} is the transition energy of 1s excitonic energy (1.65 eV), d_{1s} is the transition dipole moment of 1s exciton, and $F(t)$ is the temporal profile of the driving field. Here, the light-matter coupling is considered within the dipole-approximation, and d_{1s} is a real number for simplicity. Then, the temporal evolution of the density matrix obeys the so-called optical Bloch equations:

$$\hbar \frac{\partial}{\partial t} \rho_{0,0}(t) = 2\hbar\Omega_R(t) Im[\rho_{1s,0}^*(t)]\,, \tag{S4}$$

$$\rho_{1s,1s}(t) = 1 - \rho_{0,0}(t), \tag{S5}$$

$$\hbar \frac{\partial}{\partial t} \rho_{1s,0}(t) = (-i\varepsilon_{1s} - \hbar\gamma)\rho_{1s,0}(t) - i\hbar\Omega_R(t)(\rho_{0,0}(t) - \rho_{1s,1s}(t)), \tag{S6}$$

$$\rho_{0,1s}(t) = \rho_{1s,0}^*(t), \tag{S7}$$

Here, $\Omega_R(t) = d_{1s}F(t)$, ρ_{ij} is the *(i,j)* component of the density matrix, and γ is a phenomenological damping constant. The induced polarization $P(t)$ is given by

$$P(t) = 2d_{1s}\mathrm{Re}[\rho_{1s,0}(t)]. \tag{S8}$$

The nonlinear emission spectrum is given by $\omega^2\left|\tilde{P}(\omega)\right|^2$, where $\tilde{P}(\omega)$ is the Fourier transformation of $P(t)$. In the numerical simulation, we used Gaussian pulse given by

$$F(t) = F_0 \exp(-t^2/\Delta t^2) \cos \Omega t. \tag{S9}$$

We set the parameters as $\varepsilon_{1s} = 1.650$ eV, $\hbar\Omega = 0.258$ eV, $\hbar\gamma = 0.020$ eV, $\Delta t = 36$ fs. Here, the Rabi energy is given by $\hbar\Omega_R = d_{1s}F_0$, and the initial conditions are $\rho_{0,0} = 1$ and $\rho_{1s,1s} = \rho_{1s,0} = 0$. Figure S4(b) shows a typical nonlinear emission spectrum calculated using the two-level model with $\hbar\Omega_R = 0.6$ eV, corresponding to $1.2F_c$ in the main text. In addition to the high harmonic generation, strong exciton emission is observed. Figure S4(c) shows the temporal evolution of the polarization obtained by the Gabor transformation.

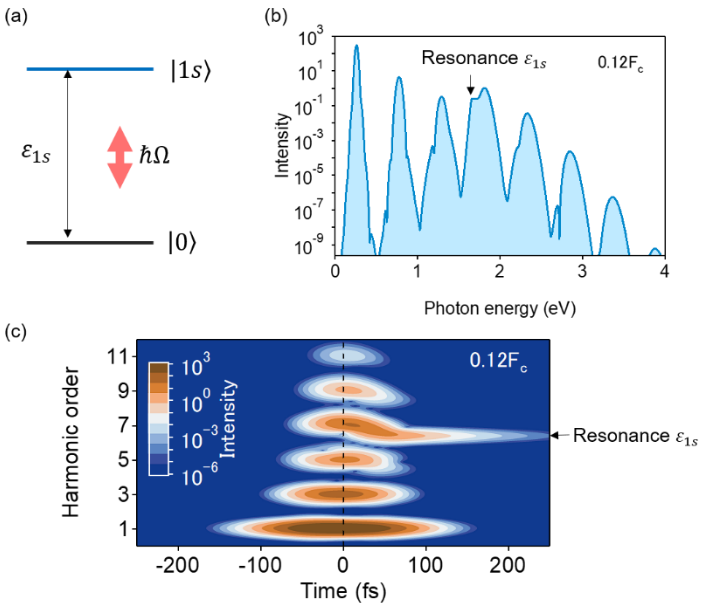

Figure S4: Numerical result of two-level dynamics. (a) The energy diagram of two-level model consisting of vacuum and 1s exciton levels. (b) The calculated nonlinear emission spectra by using two level model. The arrow indicates the 1s exciton transition energy. (c) Temporal evolution of nonlinear emission obtained by the Gabor transformation with the time window of 20 fs (FWHM).

After the peak of driving pulse ($t > 0$), the seventh harmonic signal is gradually converted into the bare excitonic emission.

S5-2. Floquet states in a two-level model.

When the driving field is a continuous wave $F(t) = F_0 \cos \Omega t$, we can apply the Floquet theorem (5), and the solution of the time-dependent Schrödinger equation can be written by

$$\Phi_\alpha(t) = e^{-\frac{i\varepsilon_\alpha^*}{\hbar}t} \sum_l \phi_\alpha^{(l)} e^{-il\Omega t}, \tag{S10}$$

$$\phi_\alpha^{(l)} = a_{\alpha,0}^{(l)} \varphi_0 + a_{\alpha,1s}^{(l)} \varphi_{1s}, \tag{S11}$$

where ε_α^* is quasienergy, $\phi_\alpha^{(l)}$ is lth sidebands of the Floquet state $\Phi_\alpha(t)$. $\phi_\alpha^{(l)}$ can be described as a superposition of bare states φ_β with a coefficient $a_{\alpha,\beta}^{(l)}$.

The set of a quasienergy ε_α^* and the coefficients $a_{\alpha,\beta}^{(l)}$ can be obtained by solving the static problem as follows:

$$\sum_m \left(H^{(l-m)} - m\Omega\delta_{lm}\right) \phi_\alpha^{(m)} = \varepsilon_\alpha^* \phi_\alpha^{(l)}, \tag{S12}$$

$$\sum_l H^{(l)} e^{-il\Omega t} = H_0 + \frac{\Omega_R}{2}\left(e^{i\Omega t} + e^{-i\Omega t}\right) \begin{pmatrix} 0 & 1 \\ 1 & 0 \end{pmatrix}, \tag{S13}$$

In the calculation, we numerically solved Eq. (S12) by considering the Fourier component of $-100 \le m, l \le 100$. Figure S5(a) shows the numerically

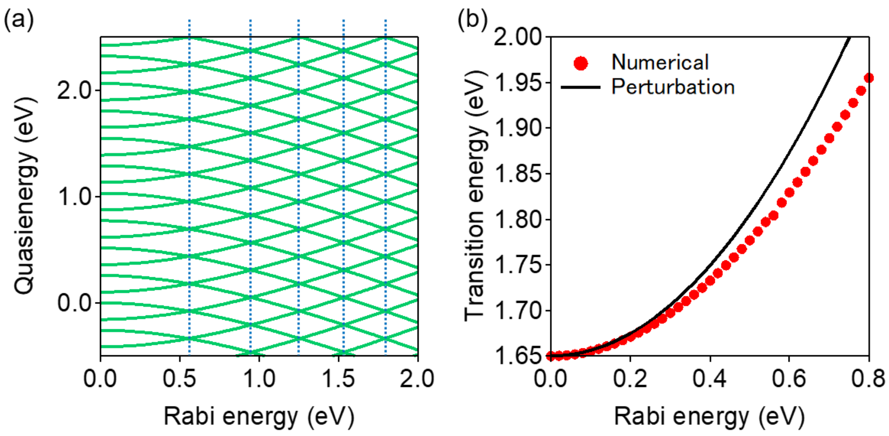

Figure S5: Quasienergy of the two-level model. (a) Numerically obtained quasienergy diagram of the two level model. Here, $\varepsilon_{1s} = 1.65$ eV and $\hbar\Omega = 0.258$ eV. The dashed lines indicate Rabi energies corresponding to the avoided crossing. (b) Transition energy shifts between two levels as a function of Rabi energy. Red circles indicate the numerically obtained one by neglecting avoided crossing, and black solid line indicates analytical one by considering up to the second-order perturbation of the interaction Hamiltonian. This energy shift within the perturbation theory corresponds to so-called ac-Stark shift and Bloch-Siegert shift.

calculated quasienergy diagram as a function of the Rabi energy (field amplitude). With the increase of Rabi energy, quasienergy is monotonically shifted as shown in Fig. S5(b). Here, we neglect the avoided crossing of quasienergy, and trace the energy with the diabatic picture. This energy shift considerably deviates from that of the perturbation-theory calculation including ac-Stark shift and Bloch-Siegert shift typically above $\hbar\Omega_{\mathrm{R}} = 0.2$ eV. The intersection between Floquet sidebands, which actually shows an avoided crossing, represents the multi-photon resonance conditions in the Floquet system given by Eq. (1) in the main text. In our experimental setup, the first avoided crossing at Rabi energy around 0.6 eV satisfies the seven-photon resonance condition. This energy corresponds to the field amplitude where excitonic coherence is efficiently created as shown in Figs. S4(b) and (c).

S5-3. Temporal evolution of Floquet state

Let us consider the dynamics of the system under temporally varying envelope $F_0(t)$ using instantaneous Floquet basis. Here, we assume that the quantum state at time t_0 can be described by the Floquet state under the constant driving amplitude $F_0(t_0)$ (instantaneous Floquet state) (*13,15*), and the transition between different instantaneous Floquet states only occurs at the avoided crossing of quasienergies. The system is in the ground state (vacuum state) at the initial time. Then, with an increase of the field amplitude, the ground state becomes the Floquet ground state as follows:

$$\Psi(t) = \Phi_{\mathrm{g}*}(t) = e^{-i\theta_{g*}(t)} \sum_l \phi_{g*}^{(l)} \left(F_0(t)\right) e^{-il\Omega t}, \tag{S14}$$

$$\theta_{g*}(t) = \int_0^t dt' \frac{\varepsilon_g^*\left(F_0(t')\right)}{\hbar}. \tag{S15}$$

Then, the induced electric polarization is given by

$$P(t) = -\langle \Psi(t) | e\hat{x} | \Psi(t) \rangle$$

$$= d_{1s} \sum_{l,m} \left[\mathrm{a}_{g*,0}^{(l)*}(F_0(t)) \mathrm{a}_{g*,1s}^{(m)}(F_0(t)) e^{i(l-m)\Omega t} + c.c. \right]$$

$$= 2d_{1s} \sum_{n,m} A_{g*,n,m}(F_0(t)) \cos(n\Omega t + \psi_{g*,n,m}(F_0(t))), \quad \text{(S16)}$$

$$A_{g*,n,m}(F_0(t)) = \left| \mathrm{a}_{g*,0}^{(n+m)*}(F_0(t)) \mathrm{a}_{g*,1s}^{(m)}(F_0(t)) \right|, \quad \text{(S17)}$$

$$\psi_{g*,n,m}(F_0(t)) = \arg \left[\mathrm{a}_{g*,0}^{(n+m)*}(F_0(t)) \mathrm{a}_{g*,1s}^{(m)}(F_0(t)) \right]. \quad \text{(S18)}$$

Equation (S16) indicates that the induced electric polarization has integer multiple frequency components with slowly varying amplitude and phase, i.e., high harmonic emissions.

When the peak field amplitude is below the first avoided crossing, the quantum state returns to the ground state, and only high harmonic emission with slight modulation can be observed. On the other hand, when the multi-photon resonance condition of the Floquet state is satisfied, the diabatic transition between ground and excited Floquet states efficiently occurs (*13-16*).

Thus, the quantum state after the transition becomes a superposition of Floquet ground and excited states as follows:

$$\Psi(t) = c_{g*} \, \Phi_{\mathrm{g*}}(t) + c_{e*} \, \Phi_{\mathrm{e*}}(t). \quad \text{(S19)}$$

Then, the induced electric polarization is given by

$$P(t) = 2d_{1s} \sum_{\alpha,n,m} |c_\alpha|^2 A_{\alpha,n,m} \cos(n\Omega t + \psi_{\alpha,n,m})$$

$$+ d_{1s} \left[c_{g*}^* c_{e*} e^{i\Delta\theta} \sum_{n,m} \left(\mathrm{a}_{g*,0}^{(n+m)*} \mathrm{a}_{e*,1s}^{(m)} + \mathrm{a}_{g*,1s}^{(n+m)*} \mathrm{a}_{e*,0}^{(m)} \right) e^{in\Omega t} + c.c. \right],$$

(S20)

where $\Delta\varepsilon^*(F_0(t))$ and $\Delta\theta(F_0(t))$ are the transition energy and phase difference between the two Floquet states, respectively. The first term describes high harmonic emissions as previously discussed. On the other hand, the second term has an extra fast-oscillating phase $\Delta\theta(F_0(t))$, which corresponds to the

frequency of $\Delta\varepsilon^*/\hbar$ at time t. Especially, when the field amplitude decreases, absolute values of $a_{g*,0}^{(0)}$ and $a_{e*,1s}^{(0)}$ becomes much larger than those of other coefficients $a_{\alpha,\beta}^{(l)}$. Therefore, at each instant of time t, the second term is approximately written by

$$d_{1s}\left[c_{g*}^* c_{e*}\left(a_{g*,0}^{(0)*}a_{e*,1s}^{(0)}\right)e^{-\frac{i\Delta\varepsilon^*(F_0(t))}{\hbar}t+const.}+c.c.\right]. \quad \text{(S21)}$$

This means that excitonic coherence with the transition energy of Floquet state $\Delta\varepsilon^*(F_0(t))$ is created after the transition. Finally, after the pulse duration, $a_{\alpha,\beta}^{(l)}$ becomes zero except for $a_{g*,0}^{(0)} = a_{e*,1s}^{(0)} = 1$. Then the induced electric polarization after pulse duration is given by

$$P(t) = d_{1s}\mathrm{Re}\left[c_{g*}^* c_{e*} e^{\frac{i\varepsilon_{1s}}{\hbar}t+const.}\right]. \quad \text{(S22)}$$

This means that the emission with the 1s exciton resonance energy can be observed after the pulse duration.

According to the above description, let us consider our experimental condition, i.e., the transition energy of the Floquet state at the peak field amplitude is almost resonant with the seventh order harmonic energy. Before the peak of the driving pulse, high harmonic emission can be observed as in Eq. (S16). Then, around the peak of the driving pulse, the dressed excitonic coherence oscillating with the seventh order harmonic energy ($\Delta\varepsilon^* = 7\hbar\Omega$) is created. With a decrease of driving field amplitude, dressed excitonic coherence energy

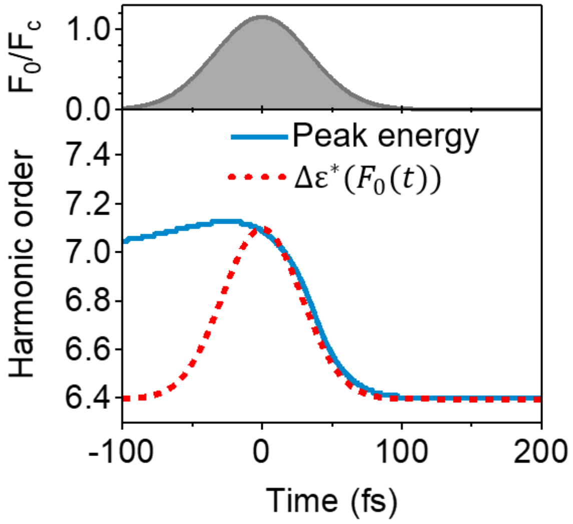

Figure S6: Dynamics of quasienergy and coherent emission. Upper panel: temporal profile of the driving field amplitude. Lower panel: Temporal evolutions of transition energy and emission energy in the vicinity of 1s excitonic resonance ε_{1s} (~6.4 in the figure). Blue solid curve indicates the peak energy of emission extracted from the Gabor transformation of polarization $P(t)$ in Fig. S4(c). The slight deviation from the seventh harmonic emission energy before the peak of the pulse is maybe due to the phase ψ_{g*} in Eq. (S16). Red dashed curve indicates the transition energy of Floquet state $\Delta\varepsilon^*(F_0(t))$. According to the time window of Gabor transformation of 20 fs, the transition energy is also averaged over 20 fs in

gradually approaches the bare 1s exciton energy according to $\Delta\varepsilon^*(F_0(t))$. Figure S6 shows the time evolutions of transition energy $\Delta\varepsilon^*(F_0(t))$ (red dashed line) and peak energy of the numerically calculated emission in the vicinity of the 1s excitonic energy. As expected, the emission energy is almost

the same as the seventh order harmonic energy before the peak, and follows $\Delta\varepsilon^*(F_0(t))$ after the peak. This indicates that our experimental condition is well described by the instantaneous Floquet states.

S5-4. Multi-photon resonance condition in the Floquet state.

To check our claim that coherence between two Floquet states is efficiently created at multi-photon resonance $\Delta\varepsilon^*(F_0, \Omega) \approx n\hbar\Omega$, we performed numerical simulations of the two-level system with the different peak amplitudes (Rabi

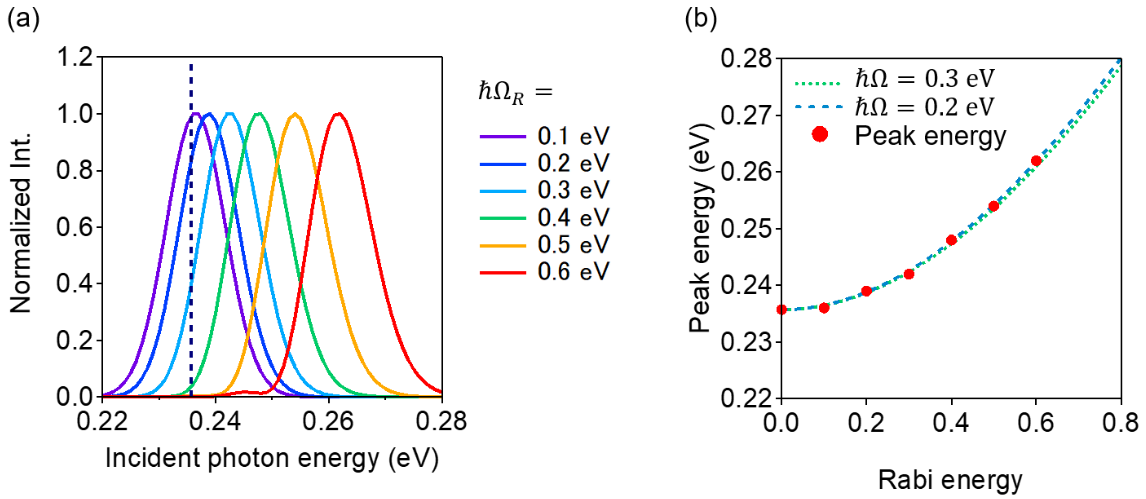

Figure S7: Seventh-photon resonance in Floquet state. (a) Emission intensities at 1.65 eV (1s excitonic resonance) as a function of driving photon energy. The intensities are normalized by the local maximum value near the seven-photon resonance. Black dashed line indicates seven-photon resonance of bare 1s exciton ($\hbar\Omega = \varepsilon_{1s}/7$). To separate the resonant emission component from the seventh order harmonic, we use Gabor window centered at 230 fs with 20 fs width, which is sufficiently after the pulse duration. (b) Red line: peak energy extracted from (a). Green dotted and blue dashed lines indicate the transition energies of the Floquet state divided by 7

energy) and driving photon energies. Figure S8(a) shows driving photon energy dependence of the resultant emission intensity resonant with the bare transition energy. When $\hbar\Omega_R$ is equal to 0.1 eV, the intensity becomes maximum almost at the seven-photon resonance of the bare 1s excitonic energy $\hbar\Omega = \varepsilon_{1s}/7$ (dashed line in Fig. S7(a)). With an increase of the peak field amplitude (Rabi

energy), the peak energy is shifted toward higher energy. At $\hbar\Omega_R = 0.6$ eV, the peak energy is almost 30 meV higher than the original seven-photon resonance. Figure S7(b) shows the driving photon energy where the resonant emission takes the maximum value as a function of Rabi energy. The peak energy (red circles) follows the transition energies of the Floquet state (green dotted line: $\hbar\Omega = 0.3$ eV, blue dashed line: $\hbar\Omega = 0.2$ eV) well. This clearly indicates the multi-photon resonance condition for Floquet state described in Eq. (1) in the main text.

S5-5. Effect of decoherence on the exciton emission.

Here, we verify the effect of decoherence on the exciton emission. Figure S8(a) shows the exciton emission and the seventh harmonic intensity as a function of the dephasing rate $\hbar\gamma$. With an increase of the dephasing rate, both the seventh harmonic and the exciton emission rapidly decrease. The decreasing rate of the exciton emission is larger than that of the seventh harmonic. Figure S8(b) shows the intensity ratio of the exciton emission to the seventh harmonic. To observe the exciton emission comparable to the seventh harmonic, the dephasing rate should be as small as possible. The dephasing rate of 1s A-exciton in WSe_2 is estimated to be around 20 meV even at room temperature. This small dephasing rate enables us to observe exciton coherence created through Floquet state formation in our experimental condition.

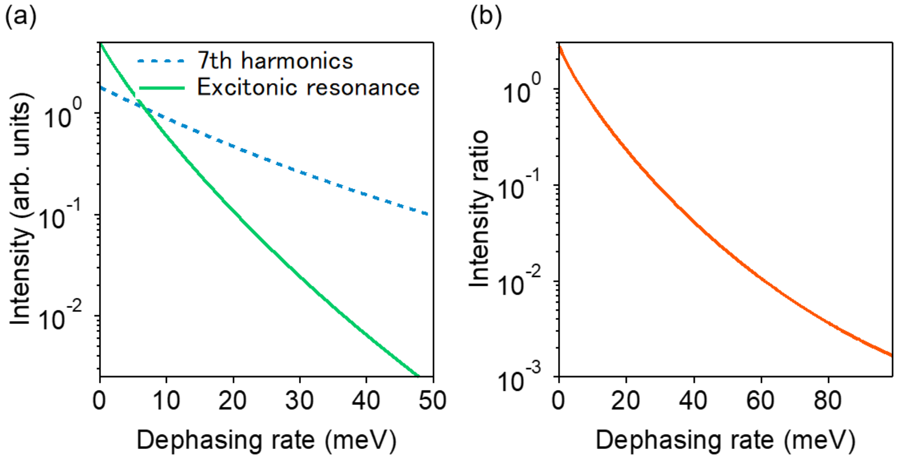

Figure S8: Dephasing effect on coherent emission. (a) Emission intensities at 1.65 eV (1s excitonic resonance: green solid line) and 1.81 eV (seventh harmonic: blue dashed line) as a function of dephasing rate $\hbar\gamma$. The intensities are taken from the peak value of Gabor transformation of polarization at each photon energy. (b) Intensity ratio of the excitonic emission to the seventh harmonic as a function of dephasing rate.

S5-6. Two-band model with excitonic effect.

Here, we consider the two-band model with excitonic effect in two-dimensional momentum space. The treatment is basically the same as Refs. (*45*) and (*46*). We numerically solved the linearized semiconductor Bloch equations as follows (*47*):

$$\frac{d}{dt}\tilde{P}_{\boldsymbol{k}}(t) = -\gamma\tilde{P}_{\boldsymbol{k}}(t) - \frac{i}{\hbar}\varepsilon_g(\boldsymbol{k} - e\boldsymbol{A}(t)/\hbar)\tilde{P}_{\boldsymbol{k}}(t) - i\Omega_{\mathrm{R},\boldsymbol{k}}(t)(2\tilde{n}_{\boldsymbol{k}}(t) - 1) + \sum_{\boldsymbol{q}\neq\boldsymbol{k}} V_{\boldsymbol{k}-\boldsymbol{q}}\tilde{P}_{\boldsymbol{q}}(t)\ , \tag{S23}$$

$$\frac{d}{dt}\tilde{n}_{\boldsymbol{k}}(t) = -\frac{2}{\hbar}Im[\Omega_{R,\boldsymbol{k}}(t)\tilde{P}^*_{\boldsymbol{k}}(t)] \tag{S24}$$

$$\boldsymbol{A}(t) = -\int dt\,\boldsymbol{F}(t) \tag{S25}$$

Here, $\tilde{P}_{\boldsymbol{k}}(t)$ and $\tilde{n}_{\boldsymbol{k}}(t)$ are the pair function and population at crystal momentum $\boldsymbol{k}$, respectively. $\varepsilon_g(\boldsymbol{k})$ and $\Omega_{\mathrm{R},\boldsymbol{k}}(t)$ are crystal-momentum

dependent bandgap energy and Rabi energy, respectively. For simplicity, we used two-dimensional cosine bands $\varepsilon_g(\boldsymbol{k}) = \varepsilon_g(\boldsymbol{0}) + t_r(1 - \cos k_x a \cos k_y a)$ ($\varepsilon_g(\boldsymbol{0}) = 1.89$ eV: bandgap energy, $\mathrm{t_r} = 3.5$ eV: hopping energy, $a = 3.3 \times 10^{-10}$ m: lattice constant), and neglected momentum dependence of Rabi energy ($\Omega_{\mathrm{R},\boldsymbol{k}}(t) = \Omega_{\mathrm{R}}(t) = d_{1s}F(t)$). $V_{\boldsymbol{k}-\boldsymbol{q}}$ is the two-dimensional Coulomb potential between electrons at $\boldsymbol{k}$ and $\boldsymbol{q}$. We set the amplitude of $V_{\boldsymbol{k}-\boldsymbol{q}}$ so that 1s exciton energy obtained by solving the Wannier equation is the same as the experimentally obtained one (1.65 eV). Here, $\Omega_{\mathrm{R},\boldsymbol{k}}(t)$ describes the creation and annihilation of electron-hole pairs, and $\varepsilon_g(\boldsymbol{k} - e\boldsymbol{A}(t)/\hbar)$ describes kinetic energy induced by the intraband motion of electron-hole pairs. To check the effect of intraband electron-hole motion on the excitonic response, we introduce a scaling parameter β in Eq. (S23) as $\varepsilon_g(\boldsymbol{k} - e\beta\boldsymbol{A}(t)/\hbar)$. β determines the strength of intraband electron-hole motion respective to the strength of interband mixing described by Rabi energy. In the numerical simulation, we used a 100×100 k-mesh and calculated polarization $P(t) = d_{1s}\sum_{\boldsymbol{k}} Re[P_{\boldsymbol{k}}(t)]$. Figure 9(a) shows the absorption spectrum obtained by solving Wannier equation. Figure S9(b) shows the corresponding nonlinear emission spectra with several β and Rabi energy of Ω_{R}~0.6 eV. When $\beta = 1$, the typical kinetic energy of electron-hole pairs $\langle \varepsilon_g(e\beta\boldsymbol{A}(t)/\hbar)\rangle_t$ is estimated to be around 1.5 eV, which is larger than the Rabi energy, and the exciton emission is strongly suppressed. This might be because strong intraband driving of electron-hole pairs causes transition of 1s exciton into higher excitonic states and unbound electron-hole pairs. In contrast, when $\beta = 0$ (without intraband driving), strong exciton emission is observed as in the two-level model. Our experimental observation, which is similar to that with $\beta \sim 0.3$, suggests that the kinetic energy of electron-hole pairs is smaller than the Rabi energy and the excitonic response can be described by a two-level system. The more refined studies, including the real electronic structure, momentum-dependent Rabi energy, and scattering process, are needed to understand why we can regard excitons in monolayer WSe_2 as a two-level system well.

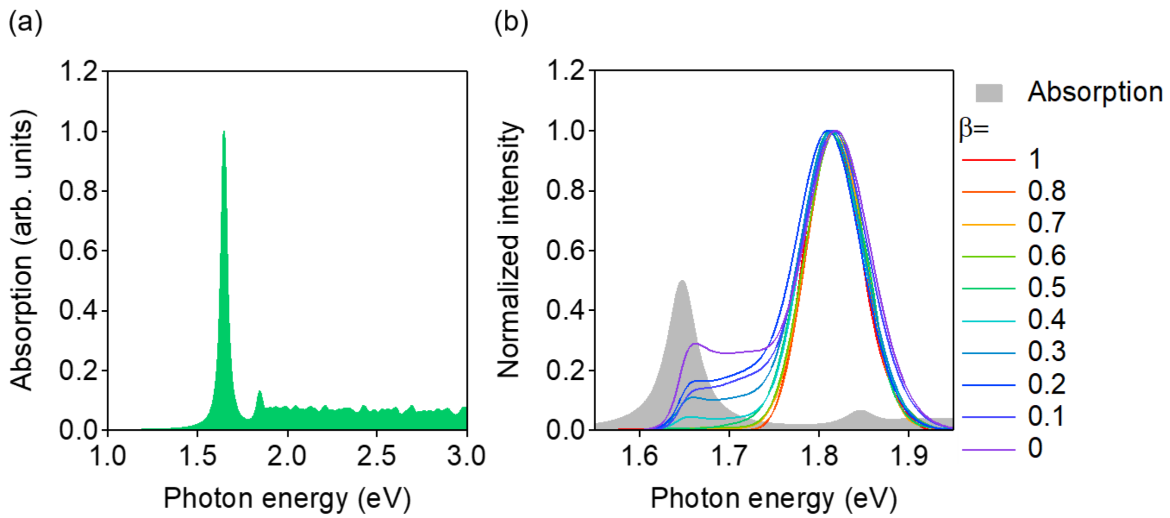

Figure S9: Nonlinear emission in two-band model. (a) Calculated absorption spectrum in two-dimensional cosine band with excitonic effects. Here, we solved Wannier equation with 100×100 k-mesh. (b) Nonlinear emission spectra near the excitonic resonance. Here, we set $\Omega_R = 0.65$ eV. Grey shaded area is the corresponding absorption spectrum.

S6. Field strength dependence of coherent excitonic signals

Here, we discuss the field strength dependence of coherent excitonic signal intensity. When we use the simple two-level model in Sec. 6-1, excitonic signal has a maximum value just above the critical field F_c ($\sim 1.2 F_c$), then shows oscillation depending on driving field strength as shown in Fig. S10. These behaviors are well explained by Floquet-Landau-Zener interference discussed in Ref. (*16*). Figures S11(a) and (b) show the seventh harmonic (green squares) and coherent exciton emission (blue circles) as a function of MIR intensity for electric field along armchair (a) and zigzag (b) directions, respectively. The

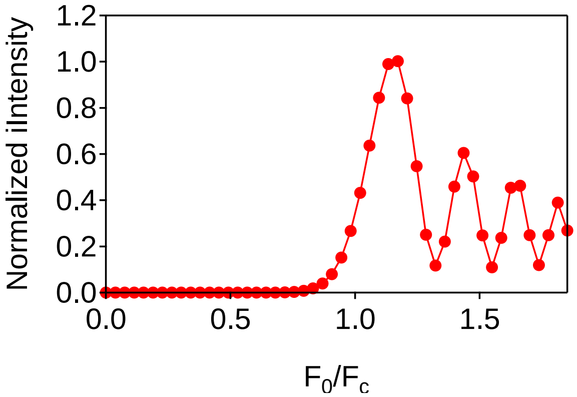

Figure S10: Calculated coherent emission intensity as a function of field strength using two-level model.

seventh harmonic signals increase monotonically with increasing MIR intensity, whereas coherent excitonic signal shows strong saturation above 100 GW/cm^2. This saturation behavior seemingly corresponds to the Floquet-Landau-Zener interference effect, but MIR intensity dependence is much smoother than that of two-level model (dashed curves). This implies that the effects neglected in the simple two-level model contribute to the experimental results although the two-level model provides us the qualitative understanding of the observation.

To investigate the discrepancy between the simple two-level model and the experimental results, we firstly checked the result of semiconductor Bloch equation (SBE) discussed in Sec. 6.5, which includes the contribution of higher excitonic levels and continuum states. Here, the parameter β introduced in Sec. 6-5 was optimized to be 0.3 so that the nonlinear emission spectrum near the exciton resonance is in best agreement with the observations. Figure S12(a) shows the result of SBE as a function of the square of Rabi energy (blue circles). SBE results has a good agreement with the two-level model ones below the Rabi energy at which excitonic signal has a maximum value. On the other hand,

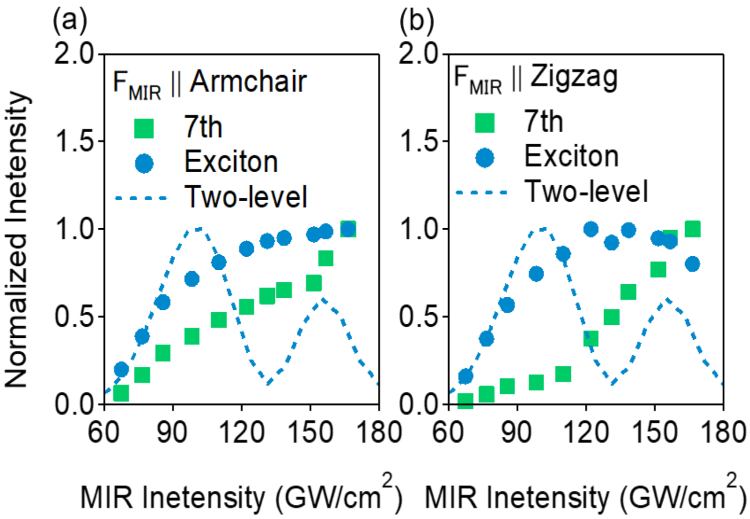

Figure S11: MIR intensity dependence of coherent emission. (a,b) Nonlinear emission intensities as a function of MIR intensity for field along armchair (a) and zigzag (b) directions. Blue circles and green squares indicate the coherent excitonic emission and the seventh harmonics, respectively. Blue dashed lines indicate resonant coherent emission calculated by using two-level model as in Fig. S10.

oscillation behavior of SBE simulation at higher field strength is relatively weak compared with the two-level model, and SBE simulation improves the difference from the observations. This implies the effect of higher excitonic states on 1s coherent exciton emission process although Floquet state dynamics is qualitatively reproduced by the simple two-level model. With an increase of the driving field strength, the created 1s-excitonic coherence is transferred into the higher excitonic levels or continuum states through intra-excitonic transitions (*51*). This increases the effective decoherence of 1s exciton and disrupts the interference between two quantum paths in Floquet-Landau-Zener interference, resulting in the weakened oscillation behavior.

We further consider the extrinsic effect on the observations: non-uniform MIR field distribution in the real experiment. Since MIR field strength distribution is not uniform, but rather close to a Gaussian distribution in our experiment, averaging might smooth out the intrinsic steep response. We summed the computed polarization density $P(t)$ for given MIR field strength over the MIR spot as follows:

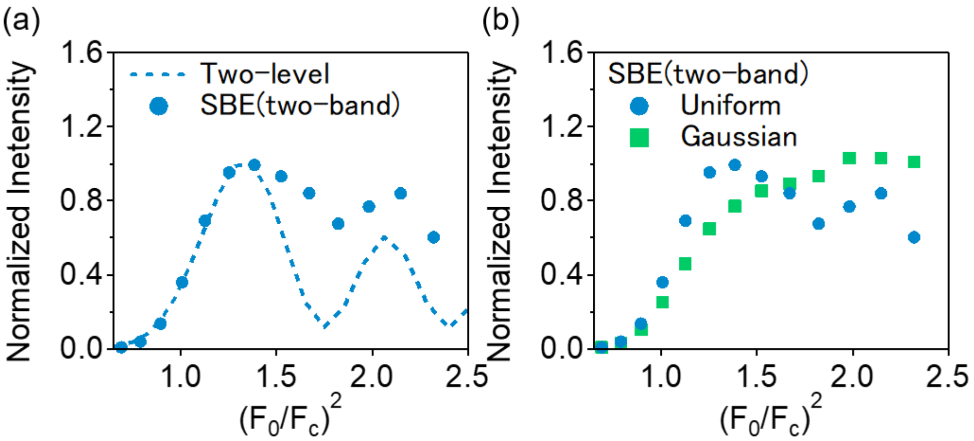

Figure S12: MIR intensity dependence of coherent emission beyond two-level model. (a,b) Calculated exciton emission intensities as a function of the square of field strength. Blue-dashed line in (a) indicates the result obtained by solving time-evolution of two-level model with uniform MIR field distribution. Blue circles indicate the result obtained by solving semiconductor Bloch equations (SBE) with uniform field distribution. Green squares indicate the SBE result with two-dimensional Gaussian distribution of MIR field.

$$P_{\mathrm{tot}}(F_0) = \int dxdyP(F(x,y)). \tag{S24}$$

Here, we assumed that MIR field distribution is a two-dimensional Gaussian with a peak field strength of F_0 given by

$$F(x,y) = F_0 \exp(-\frac{x^2+y^2}{w^2}) . \tag{S25}$$

This assumption leads to the simple form as follows:

$$P_{\mathrm{tot}}(F_0) \propto \int_0^{F_0} \frac{dFP(F)}{F} . \tag{S26}$$

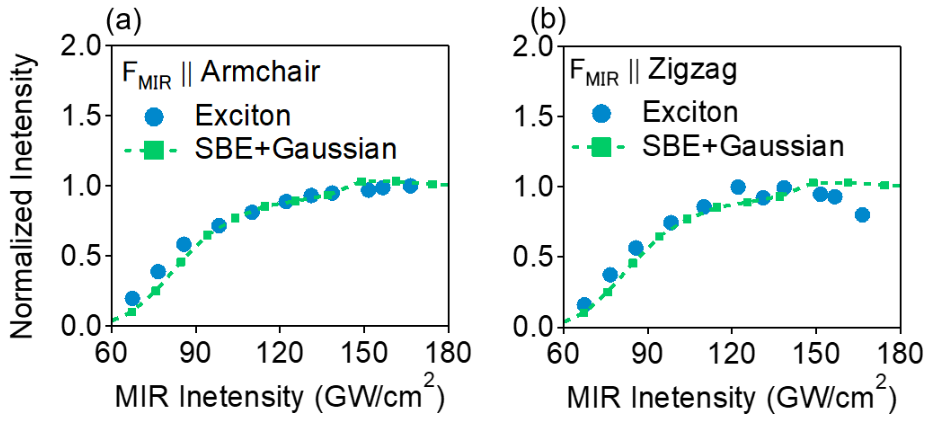

Figure S13: Comparison between experiment and simulation. Blue circles represent coherent exciton emission intensities as a function of MIR intensity for field along armchair (a) and zigzag (b) directions. Green square-dashed lines indicate the simulation results using SBE with two-dimensional Gaussian distribution. Here, the horizontal scale of the simulation results is magnified so that the simulation has best agreement with our experiment.

Figure S12(b) shows the SBE results with uniform (blue circles) and Gaussian (green squares) MIR field distribution. As expected, MIR intensity dependence with Gaussian distribution shows much smoother changes compared with that with the uniform distribution, and oscillation behavior is smeared out. Figures S13(a) and (b) show the comparison of experiments and SBE simulations including the inhomogeneous field distribution. Simulation result has an excellent agreement with the experimental observations. This means that spatial-filtering technique (*53*), which measured the nonlinear emission from the region where the driving field strength can be regarded as uniform, enables us to observe a much clearer signature of Floquet-Landau-Zener interference effect.

S7. Delayed response of electric polarization at resonance condition.

Here, we discuss the envelope of the electric polarization depending on incident frequency. For simplicity, we consider linear electric polarization described as follows:

$$P(t) = \int_{-\infty}^{t} dt' \, \chi(t - t') F(t'). \tag{S27}$$

As a response function, we consider the oscillator with exponential decay given by

$$\chi(t) = A e^{-\gamma t} \sin \omega_0 t, \tag{S28}$$

where A, ω_0, and γ are the amplitude, resonant frequency, and damping constant of the oscillator, respectively. Here, we introduce the incident electric field given by

$$F(t) = F_0 \theta(t) \sin \omega t, \tag{S29}$$

$$\theta(t) = \begin{cases} 0 & (t < 0) \\ 1 & (t \geq 0) \end{cases}. \tag{S30}$$

Then, the resultant polarization is given by

$$P(t) = -\frac{AF_0}{2((\omega_0 - \omega)^2 + \gamma^2)} [\gamma(\cos \omega t - e^{-\gamma t} \cos \omega_0 t) - (\omega_0 - \omega) \times$$

$$(\sin \omega t - e^{-\gamma t} \sin \omega_0 t)] + \frac{AF_0}{2((\omega_0 + \omega)^2 + \gamma^2)} [\gamma(\cos \omega t - e^{-\gamma t} \cos \omega_0 t)$$

$$-(\omega_0 + \omega)(\sin \omega t + e^{-\gamma t} \sin \omega_0 t)] \qquad (t \geq 0). \tag{S31}$$

First, let us consider the resonance condition $\omega = \omega_0$. In such case, the main contribution of the electric polarization is described by

$$P(t) \approx -\frac{AF_0}{2\gamma}(1 - e^{-\gamma t})\cos\omega t. \tag{S32}$$

As shown in Fig. S14 (solid red line), the envelope of electric polarization at the resonance condition is gradually increase with a time-scale of $1/\gamma$. Next, let us consider the non-resonance condition $\gamma \ll |\omega - \omega_0|$. This results in the electric polarization given by

$$P(t) \approx \frac{AF_0}{\omega_0^2 - \omega^2}(\omega \sin\omega t - \omega_0 \sin\omega_0 t). \tag{S33}$$

In this case, the envelope of the signal oscillates with the detuning between the oscillator resonance and incident laser frequency $(\omega - \omega_0)$ (see solid blue line in Fig. S10). Therefore, the time scale of the build-up of electric polarization is given by $(\omega - \omega_0)^{-1}$. This indicates that the response of the electric polarization becomes instantaneous with an increase of the detuning. This discussion can be applied to the nonlinear electric polarization, and the delayed seventh harmonic signal with respect to the envelope of the incident driving field as shown in Fig. 4(d) suggests that the seventh harmonic is resonant with the transition energy between Floquet states.

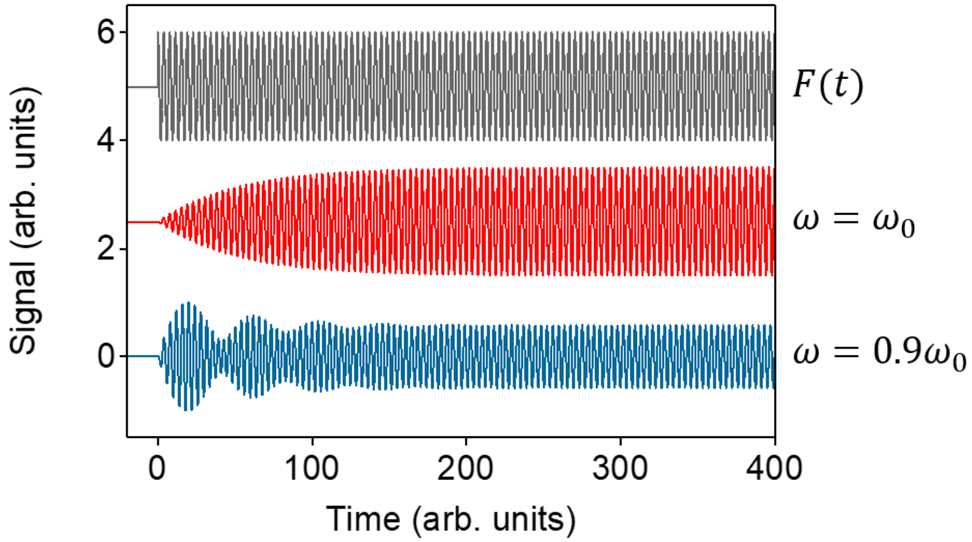

Figure S14: Electric polarization induced by the driving field with step-like envelope. Here, the parameters are set as follows: $\omega_0 = 1.65$ and $\gamma = 0.02$. Grey line indicates the temporal profile of the driving field $F(t)$. Red and blue solid lines indicate the electric polarization $P(t)$ with $\omega = \omega_0$ and $\omega = 0.9\omega_0$, respectively.
